# Mitral Valve Leaflet Repair with Autologous Saphenous Vein in a Child with Infective Endocarditis

**DOI:** 10.1007/s00246-025-03826-4

**Published:** 2025-03-14

**Authors:** Jacques T. Janson, Barend Fourie, Pierre Goussard, Helena Rabie, Jinyong Kim

**Affiliations:** 1 Division of Cardiothoracic Surgery, Faculty of Medicine and Health Sciences, University of Stellenbosch, Franci Van Zijl Drive, Tygerberg, 7505 South Africa; 2 Department of Pediatrics, Stellenbosch University, Tygerberg, South Africa

**Keywords:** Mitral valve repair, Infective edocarditis, Saphenous vein

## Abstract

Saphenous vein has been used successfully to repair the mitral valve in adults. We describe the use of a saphenous vein patch to repair a large defect in the anterior mitral valve leaflet in a child with infective endocarditis to avoid a mechanical mitral valve replacement.

**Supplementary Information:**

The online version contains supplementary material available at 10.1007/s00246-025-03826-4.

## Introduction

Valves damaged by infective endocarditis can be very challenging to repair if large parts of the valve are destroyed. Patch material that can grow and adapt with a valve leaflet does not exist currently [1–3].

Untreated fresh autologous pericardium shrinks and retracts and glutaraldehyde-treated pericardium degenerates and calcifies.

Using harvested autologous vein to replace an anterior mitral valve leaflet was described in a sheep model. In this model, the harvested jugular vein was used as a living tissue patch supported with extended polytetrafluoroethylene (ePTFE) chordae. The vein leaflet remained viable and functional as a mitral valve leaflet in the sheep for up to 10 months [4].

In humans, saphenous vein patches are used to extend the anterior mitral leaflet after debridement for infected endocarditis. The leaflet edge is supported with ePTFE chordae. The saphenous vein is autologous living tissue with an intima, media and adventitia and is strong enough to hold the ePTFE chordae [4, 5]. The patch remains flexible and mobile for up to 3 years on echocardiography and mitral valve regurgitation is prevented [4–6].

In this report we describe the first use of autologous saphenous vein patch repair of the anterior mitral leaflet in a young adolescent with severe mitral valve damage secondary to infective endocarditis.

## Report

**Fig. 1:**
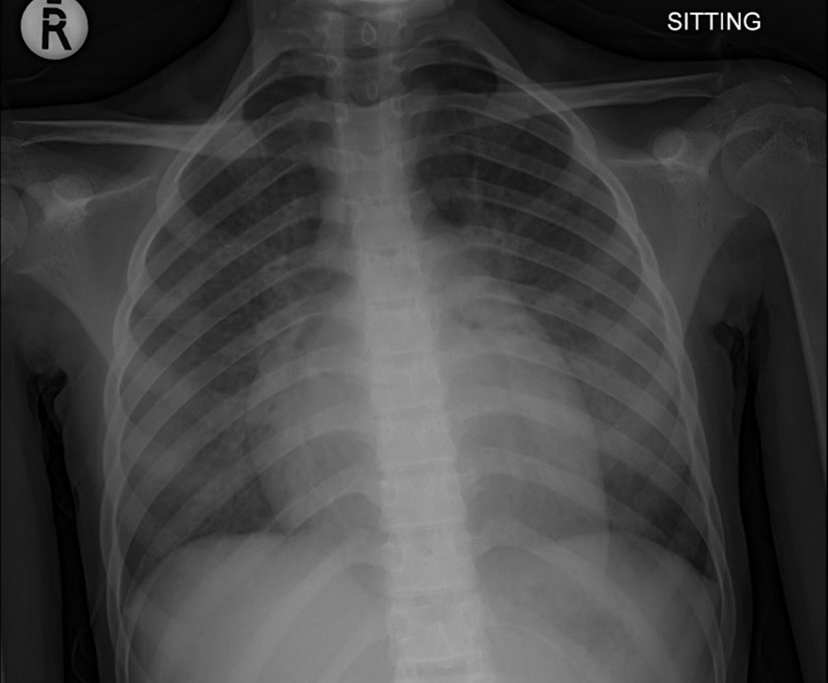
Preoperative chest x-ray showing cardiomegaly and signs of pulmonary edema. Note the splaying of the carina from the enlarged left atrium

**Fig. 2:**
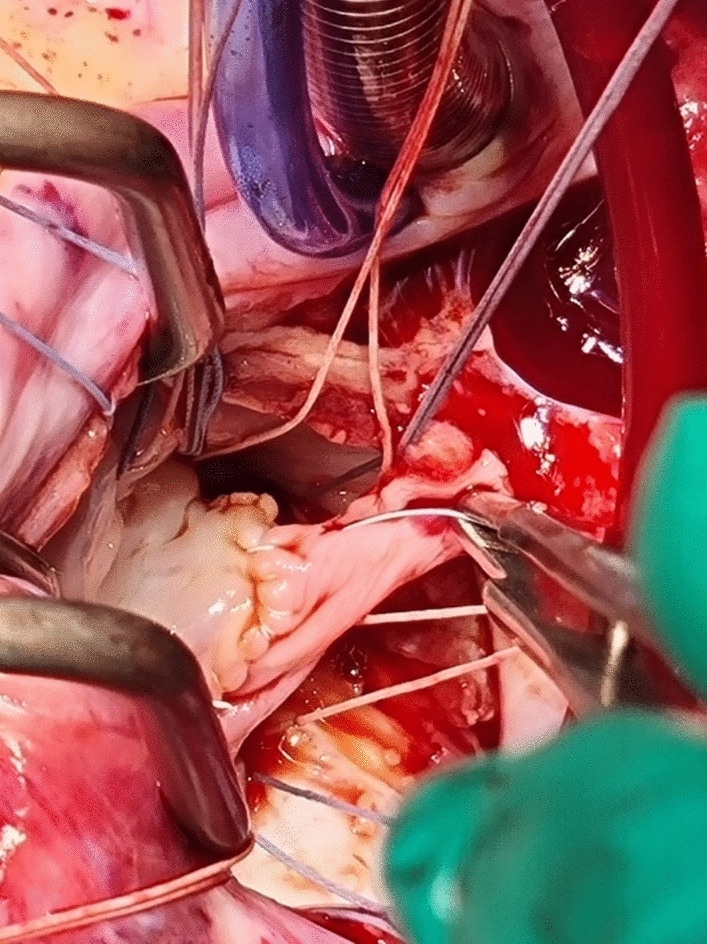
The saphenous vein sutured to the anterior leaflet remnant with Gore-Tex® CV 7

An 11-year-old adolescent girl with a weight of 33 kg and a height of 135 cm was presented to our Pediatric Cardiology department with Grade IV dyspnea and hypoxia. A mitral regurgitation murmur was heard on auscultation with crackles in the lung. The CXR showed features of pulmonary edema, with cardiomegaly and splaying of the carina from an enlarged left atrium (Fig. 1). She was diagnosed with mitral regurgitation and left-sided cardiac failure and required non-invasive ventilatory support. The echocardiography (ECHO) showed large vegetations on the anterior mitral leaflet with a flail anterior leaflet and severe MR (Fig. 2, Supplementary video 1). There was no evidence of dental caries. Three blood cultures were negative and she was treated empirically for infective endocarditis with ampicillin cloxacillin and gentamicin.

The patient and parents were counseled and consented for a mitral valve replacement with the option of a mitral valve repair if possible. Consent was also obtained to harvest a short segment of saphenous vein from the upper leg if needed.

At surgery, the mitral valve anterior leaflet had vegetations on the free edge and the chords of A1, A2, and A3 were ruptured. The whole anterior leaflet was flail with destruction of the free edge of the leaflet. The leaflet edge and chords were debrided and sent for histology, PCR, and culture.

**Fig. 3:**
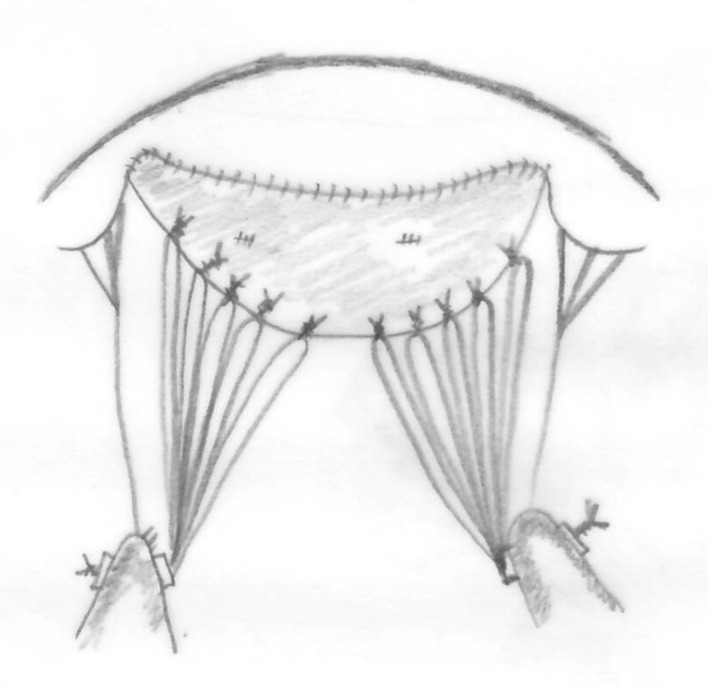
The saphenous vein patch sutured to the anterior mitral leaflet is supported with Gortex® CV 5 chordal loops from the anterior and posterior papillary muscles. Native chordae are shown to the commissural leaflets

Four centimeters of the right saphenous vein was harvested from the right upper leg and the vein was opened length wise to form a rectangular patch. The vein was bathed in a mixture of 200-ml normal saline with 4000-IU Heparin, 5-mg Verapamil, and 2-ml Sodium bicarbonate 4.2% to bring the pH to 7.3. The fresh vein was used as a patch to lengthen the free edge of the anterior mitral leaflet (Fig. 2). The intimal surface of the vein was used on the atrial side of the leaflet. This was sutured with Gore-Tex CV 7 (Fig. 3).

Gore-Tex® ePTFE chordal loops were prepared from a Gore-Tex® CV 5 suture and were pre-measured to the correct length from the anterior papillary muscle to the level of the annulus. Five chordal loops were sutured to the anterior papillary muscle and 5 to the posterior papillary muscle. Ten ePTFE chords in total supported the free edge of the vein patch. A 28-mm Medtronic® CG-Future ring was implanted to stabilize the annulus. The valve was tested with normal saline and was competent with a mild central jet.

The post-operative ECHO showed good mitral valve coaptation with only mild mitral regurgitation. (Supplementary video 2).

Postoperatively, the patient developed a left lower lobe collapse which responded to physiotherapy and mobilization. The rest of the post-operative recovery was uneventful. Culture of the valve tissue was negative but the PCR showed Streptococcus Viridans. She completed her antibiotics in hospital and was discharged 4 weeks after surgery. She will be followed up regularly.

## Comment

The saphenous vein can be used to patch a defect in the mitral valve leaflet with the vein shown to function well and remain flexible in the mitral position [6]. In this report, we demonstrated that the saphenous vein can also be used in older children and young adolescents as a patch to repair a mitral valve leaflet. This enabled us to avoid a valve replacement with a mechanical valve and its potential complications of thrombo-embolism and bleeding. Long-term anticoagulation is not necessary for a mitral valve repair with a saphenous vein patch. The saphenous vein is autologous living tissue with an intima, media, and adventitia and is strong enough to hold the ePTFE sutures. The upper leg saphenous vein is of sufficient quality to use as a vein patch in children.

Our longest follow-up in adult patients is 9 years with good function and we will closely monitor this patient to see whether the autologous vein patch will remain functional in the long term.

## Supplementary Information

Below is the link to the electronic supplementary material.

Supplementary file1 (MP4 9706 KB) Preoperative echocardiogram showing severe mitral regurgitation and flail anterior leaflet with vegetation

Supplementary file2 (MP4 842 KB) Postoperative echocardiogram showing mild mitral regurgitation after valve repair

## Data Availability

No datasets were generated or analysed during the current study.

## Abbreviations

MR: Mitral regurgitation
ePTFE: Expanded polytetrafluoroethylene
SBE: Infective endocarditis
PCR: Polymerase chain reaction

## References

[1] Hisatomi K, Isomura T, Hirano A et al (1992) Long-term follow-up results after reconstruction of the mitral valve by leaflet advancement. Ann Thorac Surg 54(2):271–275 1637217 10.1016/0003-4975(92)91382-j

[2] Shomura Y, Okada Y, Nasu M et al (2013) Late results of mitral valve repair with glutaraldehyde-treated autologous pericardium. Ann Thorac Surg 95(6):2000–2005 23622701 10.1016/j.athoracsur.2013.02.024

[3] Zaidi AH, Nathan M, Emani S et al (2014) Preliminary experience with porcine intestinal submucosa (CorMatrix) for valve reconstruction in congenital heart disease: histologic evaluation of explanted valves. J Thorac Cardiovasc Surg 148(5):2216–2225 24698560 10.1016/j.jtcvs.2014.02.081

[4] Janson JT, Coetzee A, Rossouw G et al (2017) Replacing the anterior mitral valve leaflet with autologous jugular vein in a sheep model. Ann Thorac Surg 104(2):584–592 28274518 10.1016/j.athoracsur.2016.11.060

[5] Fenoglio JJ, Pham TD, Wit AL, Bassett AL, Wagner BM (1972) Canine mitral complex: ultrastructure and electromechanical properties. Circ Res 31(3):417–430 5057021 10.1161/01.res.31.3.417

[6] Janson JT, Pecoraro A (2019) Reinventing the saphenous vein: Reconstructing the anterior mitral leaflet with a saphenous vein. Ann Thorac Surg 107(4):e287–e289 30513313 10.1016/j.athoracsur.2018.10.062

